# Characterization of a salt-induced *DhAHP*, a gene coding for alkyl hydroperoxide reductase, from the extremely halophilic yeast *Debaryomyces hansenii*

**DOI:** 10.1186/1471-2180-9-182

**Published:** 2009-08-28

**Authors:** Hsiu-fung Chao, Yung-fu Yen, Maurice SB Ku

**Affiliations:** 1 Graduate Institute of Agriculture, National Chiayi University, Chiayi, Taiwan, Republic of China 60004; 2 Graduate Institute of Agricultural Biotechnology, National Chiayi University, Chiayi, Taiwan, Republic of China 60004

## Abstract

**Background:**

*Debaryomyces hansenii *is one of the most salt tolerant species of yeast and has become a model organism for the study of tolerance mechanisms against salinity. The goal of this study was to identify key upregulated genes that are involved in its adaptation to high salinity.

**Results:**

By using forward subtractive hybridization we have cloned and sequenced *DhAHP *from *D. hansenii *that is significantly upregulated during salinity stress. *DhAHP *is orthologous to the alkly hydroperoxide reductase of the peroxiredoxin gene family, which catalyzes the reduction of peroxides at the expense of thiol compounds. The full-lengthed cDNA of *DhAHP *has 674 bp of nucleotide and contains a 516 bp open reading frame (ORF) encoding a deduced protein of 172 amino acid residues (18.3 kDa). *D. hansenii *Ahp is a cytosolic protein that belongs to the Ahp of the 1-Cys type peroxiredoxins. Phylogentically, the *Dh*Ahp and *Candida albicans *Ahp11 (Swiss-Prot: Q5AF44) share a common ancestry but show divergent evolution. Silence of its expression in *D. hansenii *by RNAi resulted in decreased tolerance to salt whereas overexpression of *DhAHP *in *D. hansenii *and the salt-sensitive yeasts *Saccharomyces cereviasiae *and *Pichia methanolica *conferred a higher tolerance with a reduced level of reactive oxygen species.

**Conclusion:**

In conclusion, for the first time our study has identified alkly hydroperoxide reductase as a key protein involved in the salt tolerance of the extremely halophilic *D. hansenii*. Apparently, this enzyme plays a multi-functional role in the yeast's adaptation to salinity; it serves as a peroxidase in scavenging reactive oxygen species, as a molecular chaperone in protecting essential proteins from denaturation, and as a redox sensor in regulating H_2_O_2_-mediated cell defense signaling.

## Background

*Debaryomyces hansenii *is an ascomycetous salt- and high pH-tolerant yeast that has been defined as halotolerant or halophilic [1]. It was isolated from saline environments such as sea water [2] or concentrated brines [3], representing one of the most salt tolerant species of yeasts. This marine yeast can tolerate salinity levels up to 24% (4.11 M) of NaCl [2]. In contrast, growth of the Baker's yeast *Saccharomyces cerevisiae *is severely inhibited when salinity reaches 10% NaCl [3]. Thus, *D. hansenii *has become a model organism for the study of salt tolerance mechanisms in eukaryotic cells [4]. It is now well recognized that the mechanisms by which all organisms achieve osmotic and ionic equilibrium are mediated by orthologous mechanisms based on conserved biochemical and/or physiological functions that are inherently necessary for essential metabolic processes [5].

Under saline conditions, *D. hansenii *accumulates large amounts of Na^+ ^without being intoxicated even when K^+ ^is present at low concentration in the environment [6]. In fact, Na^+ ^improves growth and protects *D. hansenii *in the presence of additional stress factors [1]. For example, at high or low temperature and extreme pH growth of the yeast is improved by the presence of 1 M NaCl [7]. It has been clearly shown that sodium ions are less toxic for *D. hansenii *as compared with other organisms; therefore, it is considered a 'sodium-includer' organism [8]. The reduced toxic effect by Na^+ ^and its accumulation at high levels under high salt is probably indicative of an adaptive strategy of *D. hansenii *for growth in hypersaline environments [9]. The organism must posses an array of advantageous characteristics that collectively confer its high halotolerance. Earlier studies have identified a number of salt-related genes in the extreme halophilic yeast *D. hansenii*, such as *HOG1 *(MAP kinase involved in high-osmolarity glycerol synthesis pathway) [10], *ENA1 *and *ENA2 *(plasmamembrane Na^+^-ATPase [11], *GPD1 *and *GPP *(NAD-glycerol-3-phosphate dehydrogenase and glycerol-3-phosphatase) [12], *NHX1 *(vacuolar Na^+ ^antiporter) [13] and *KHA1 *(Na^+^/H^+ ^antiporter) [14]. As expected, most of these salt-upregulated genes are involved in osmoregulation or transport of ions. However, the collective underlying mechanisms by which *D. hansenii *tolerates high levels of NaCl remain unkown.

All aerobic organisms require oxygen for efficient production of energy, but at the same time the organisms are constantly exposed to oxidative stress. This can be caused by partially reduced forms of molecular oxygen (e.g. superoxide radical, hydrogen peroxide and hydroxyl radical), known as reactive oxygen species (ROS), which are generated as normal metabolism byproducts, especially from respiration [15]. These ROS are highly reactive molecules that are capable of damaging cellular constituents such as DNA, RNA, lipids and proteins [16]. In adaptation to oxidative stress, aerobic organisms have evolved multiple enzymatic and non-enzymatic defense systems to protect their cellular constituents from ROS and to maintain their cellular redox state [17]. Accumulation of ROS is known to increase under many, if not all, stress conditions as the defensive scavenging systems become insufficient to cope with increasing levels of stress.

The enzymatic scavenging system for ROS involves a number of enzyme-catalyzed reactions in different cellular compartments. A series of peroxidases referred to as peroxiredoxins (Prxs) that are ancestral thiol-dependent selenium- and heme-free peroxidases [18] have been found from archaea, lower prokaryotes to higher eukaryotes. These peroxidases constitute a large family including bacterial AhpC proteins and eukaryotic thioredoxin peroxidases (TPxs) [19]. Prxs are abundant, well-distributed peroxidases that reduce H_2_O_2_, organic peroxides and peroxynitrite at the expense of thiol compounds. Thus, Prxs are considered alternative hydroperoxide scavenging enzymes, as they can reduce both organic and inorganic peroxides as well as oxidized enzymes. Based on the number of cysteine residues involved in catalysis, Prxs can be divided into three classes: typical 2-Cys Prxs, atypical 2-Cys prxs and 1-Cys Prxs [20]. Prxs are ubiquitous proteins that use an active site Cys residue from one of the homodimers to reduce H_2_O_2_. The peroxidative cysteine sulfenic acid formed upon reaction with peroxide is reduced directly by glutathione. It is suggested that Prxs can act alternatively as peroxidases or as molecular chaperones by changing their molecular complexes. Furthermore, the oxidized cysteinly species, cysteine sulfenic acid, may play a dual role by acting as a catalytic intermediate in the peroxidase activity and as a redox sensor in regulating H_2_O_2_-mediated cell defense signaling.

Alkyl hydroperoxide reductase (Ahp) is the second known member of a class of disulfide oxidoreductases [21] and a member of the thiol-dependent peroxiredoxin family [20], which possesses activity against H_2_O_2_, organic peroxides, and peroxynitrite [22]. Therefore, expression of *Ahp *genes plays an important role in peroxide resistance (oxidative stress) in *Bacillus subtilis *[23], *Clostridium pasteurianum *[24] and *Burkholderia cenocepacia *[25]. Moreover, the compensatory expression of *AhpC *in *Burkholderia pseduomallei katG *is essential for its resistance to reactive nitrogen intermediates [26].

In this article, we report the isolation of *DhAHP *from the extreme halophilic yeast *D. hansenii *via subtractive hybridization of cDNA isolated from high salt treated vs. non-treated cells. Further characterization of *DhAHP *showed that it is a gene orthologous to alkyl hydroperoxide reductase of *Candida albicans *(Gene ID: 3637850 AHP11). On the basis of the deduced amino acid sequence, we propose that *Dh*Ahp be classified as an alkyl hydroperoxide reductase. Silencing of its expression in *D. hansenii *by RNAi resulted in decreased tolerance while overexpression conferred enhanced tolerance to salinity. Furthermore, overexpression of *DhAHP *in the salt-sensitive *S. cerevisiae *and *P. methanolica *also endowed upon their cells greater tolerance to NaCl. These overexpression transformants exhibited reduced levels of ROS under salinity stress. These results suggest that the cytosolic Ahp, induced and accumulated under saline conditions, may play a key role in this extremely halophilic yeast in adaption to high salinity by scavenging ROS, serving as chaperone and mediating H_2_O_2_-mediated defense signaling.

## Results

### Characterization of salt-induced gene in *D. hansenii*

In this study, forward subtractive hybridization PCR was employed to investigate the genes of *D. hansenii *that are induced by salt. The subtracted cDNA library was enriched in differentially expressed sequences after treatment with 2.5 M NaCl for 24 min, relative to control cDNA. One of the selected clones that showed a significant increase in expression after salt induction is a homolog to the gene encoding for alkyl hydroperoxide reductase in *C. albicans *(Gene ID: 3637850 AHP11). This *D. hansenii *gene, *DhAHP*, was further characterized for its genomic organization, expression pattern and function.

### Cloning of full-lengthed cDNA of *DhAHP*

**Figure 1:**
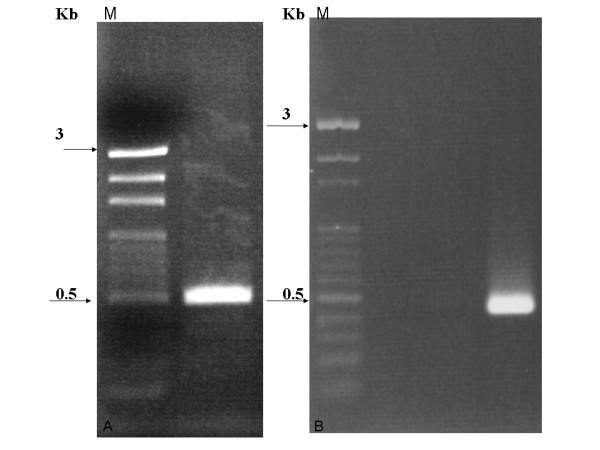
**Gel analysis of the *DhAHP *3'-end (A) and 5'-end (B) amplification products from *D. hansenii***.

To obtain a full-lengthed cDNA for *DhAHP *a forward gene specific primer (GSP) was designed and used for amplification of the 3' end of *DhAHP*, based on the partial sequence of the clone isolated from the subtracted cDNA library. A single DNA fragment of about 433 bp (Fig. 1A) was amplified using the primers of GeneRace 3' and forward GSP. According to the 3'-end fragment sequence, a specific reverse GSP was designed to amplify the 5'-end of *DhAHP *and a fragment of 557 bp was obtained (Fig. 1B). Alignment of the 3' and 5' RACE products showed that the full-lengthed cDNA of *DhAHP *has 240 bp overlapped, while 59 bp of the 5' untranslated region (UTR) is found upstream of the first ATG codon and 99 bp of the 3' UTR is found downstream from the stop codon in the amplified sequence.

**Figure 2:**
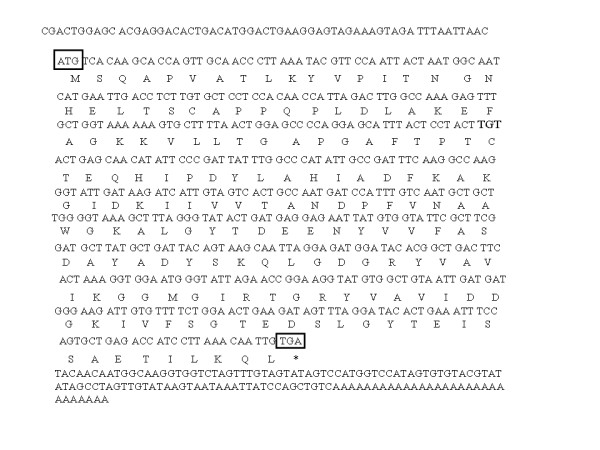
**The full-lengthed nucleotide sequence of *DhAHP *and the predicted amino acid sequence**. The start and stop codons ATG and TGA were boxed.

The full-lengthed cDNA of *DhAHP *has 674 bp of nucleotide and contains a 516 bp open reading frame (ORF) encoding a deduced protein of 172 amino acid residues (Fig. 2). The protein has an isoelectric point (pI) of 4.84 and a calculated molecular mass of 18.3 kDa. The richest amino acids are Ala (11.7% by frequency), followed by Gly (9.4%), Thr (8.8%), Asp (7.6%), Lys (7.0%), Leu (7.0%), Val (6.4%) and Ile (6.4%). Hydrophobic and hydrophilic amino acids account for 57.8% and 42.2% of the total amino acids, respectively.

### Characteristics of *DhAHP *and related genes

**Figure 3:**
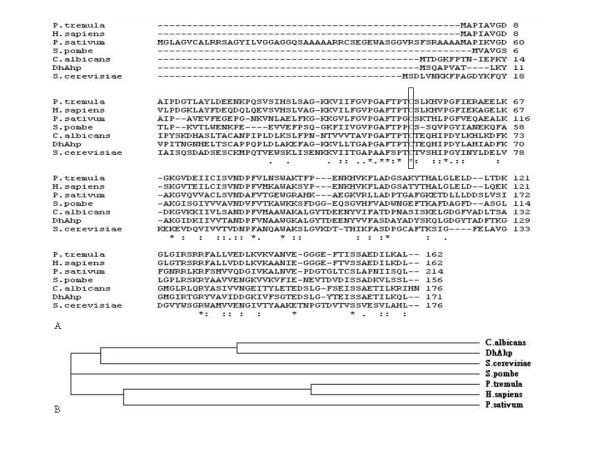
**A. Multiple alignment of related sequences to *Dh*Ahp**. The alignment was performed using the software of CLUSTAL W program http://www.ebi.ac.uk/Tools/clustalw2/index.html. Asterisks indicate identical amino acids and periods show conserved amino acid substitutions. Percent of overall identity similarity (in parentheses): 1. *Dh*Ahp; 2. AHP1 of *S. cerevisiae *(Swiss-Prot: P38013) (30%); 3. PMP20 of *S. pombe *(Swiss-Prot: O14313) (33%); 4. AHP11 of *C. albicans *(Swiss-Prot: Q5AF44) (58%); 5. peroxiredoxin of *P. tremula *(Swiss-Prot: Q8S3L0) (34%); 6. peroxiredoxin of *P. sativum *(Swiss-Prot: B3GV28) (37%); 7. peroxiredoxin of *H. sapiens *(Swiss-Prot: P30044) (25%). Cys54, conserved in all Prxs, is identified as the peroxidative cysteine. **B**. **The phylogenetic relationship between *Dh*Ahp and peroxiredoxin from other organisms**.

The deduced *D. hansenii *Ahp amino acid sequence was compared with those of related proteins from the EMBL database using the EMBOSS alignment program. The analysis showed that the protein has 72.7% similarity to *C. albicans *alkyl hydroperoxide reductase (Gene ID: 3637850 AHP11). Thus, the isolated gene is homologous to the *Ahp *gene of *C. albicans *and is therefore named *DhAHP*. The *Dh*Ahp sequence was also compared with a number of previously identified Ahp and peroxiredoxin homologs from different organisms using the protein sequence alignment program CLUSTAL W. Multiple sequence alignment analysis showed that *Dh*Ahp has 58% similarity to AHP11 (Swiss-Prot: Q5AF44) of *C. albicans*, 37% to peroxiredoxin of *Pisum sativum *(Swiss-Prot: B3GV28), 34% to peroxiredoxin of *P. tremula *(Swiss-Prot: Q8S3L0), 33% to PMP20 of *Schizosaccharomyces pombe *(Swiss-Prot: O14313), 30% to AHP1 of *S. cerevisiae *(Swiss-Prot: P38013), and 25% to *Homo sapiens *peroxiredoxin 5 (Swiss-Prot: P30044) (Fig. 3A). Furthermore, Cys-54, which is conserved in all related Prxs, is identified as the peroxidative cysteine in *Dh*Ahp.

Phylogenetic analysis revealed that the *Dh*Ahp protein is more homologous to yeast Ahps than to other Ahps from plants or peroxiredoxins from mammals. The *Dh*Ahp is located in the same subgroup as Ahps from yeasts, such as *C. albicans *and *S. cerevisiae*. Taken together, these results suggest that the Ahp of *D. hansenii *is more closely related to those of yeasts than to the plant Ahps or mammalian peroxiredoxins. It is conceivable that its function or enzymatic characteristics may be close to those of yeast Ahps (Fig. 3B).

### Genome organization and expression of *DhAHP*

**Figure 4:**
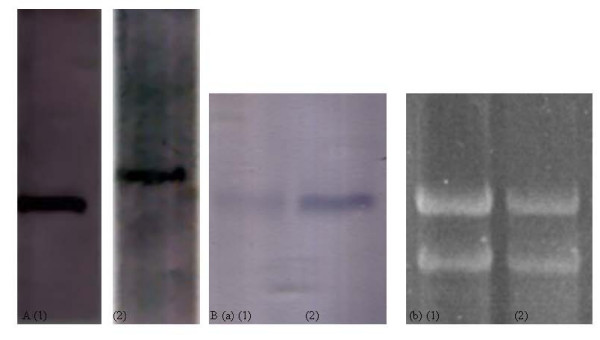
**A. Southern blot showing a single restriction fragment of *D. hansenii***. Approximately 20 μg total DNA was digested to completion with *Eco*RI (lane 1) or *Bam*HI (lane 2), electrophoresed on agarose gel, transferred to nylon membrane and hybridized to *DhAHP *probe. **B. Northern blot of *DhAHP *transcript as affected by salt treatment**. Total RNA was isolated and electrophoresed on agarose-formaldehyde gel, transferred to nylon membrane and hybridized to *DhAHP *probe (A). The gel was stained with ethidium bromide prior to blotting (B). Lane 1 and 2 indicate RNAs extracted from *D. hansenii *cells after inducted by 2.5 M NaCl for 0 and 16 min, respectively.

Southern blot analysis showed a single DNA fragment with homology to *DhAHP *(Fig. 4A) suggesting that it exists as a single copy in the genome of *D. hansenii*. Northern blot analysis revealed that expression of *DhAHP *is modulated by salt. A single transcript was detected in the cells treated with 2.5 M NaCl for 16 min (Fig. 4B). In contrast, only a small amount of the transcript was present in the control cell. Based on the differences in band intensity, it is evident that expression of *DhAHP *increased several fold only after 16 min of salt treatment. Thus, expression of the gene is rapidly induced by salt in *D. hansenii*.

**Figure 5:**
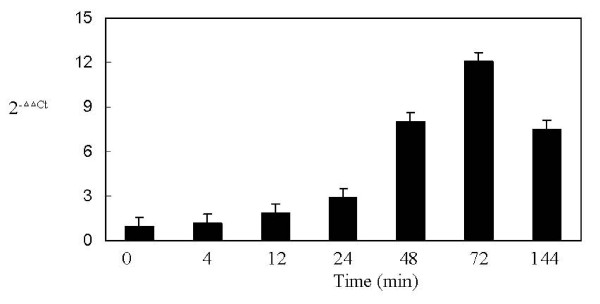
**Time course of induction of *DhAHP *transcript by 2.5 M NaCl, as determined by real-time RT-PCR**. Its transcript level increased 1.3, 1.9, 2.9, 8.0, 12.1 and 6.1 fold after 4, 12, 24, 48, 72 and 144 min of induction, respectively. Data presented were means +/- S.D. from 3–4 replicates of measurement.

The time course of induction of *DhAHP *by salt was further analyzed by relative quantification real-time RT-PCR. A small increase in *DhAHP *transcript was detected as early as 4 min upon salt (2.5 M NaCl) treatment, but its expression was rapidly accelerated thereafter. Its level increased 1.9 and 2.9 fold over the control at 12 and 24 min, respectively, with the maximum induction of 8.0 to 12.1 fold occurring between 48 and 72 min. After reaching its peak of expression at 72 min, the transcript dropped off at 144 min (Fig. 5).

### Silencing by RNA interference and overexpression of *DhAHP *in *D. hansenii*

**Figure 6:**
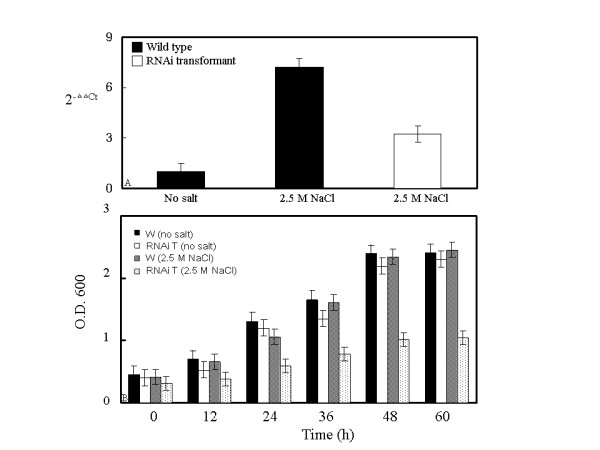
**(A) Relative levels of *DhAHP *transcript of *D. hansenii *and its RNAi transformant as affected by salt**. Cells were grown on YM11 media containing 2.5 M NaCl for 72 min, and their *DhAHP *transcripts determined by real-time RT-PCR. **(B) Growth of *D. hansenii *and its *DhAHP *RNAi transformant**. Cells were grown on YM11 media with or without 2.5 M NaCl. W: wild type strain, RNAi T: RNAi transformant. Data presented were means +/- S.D. from 3–4 replicates of measurement.

To assess the effect of loss-of-function and gain-of-function of *DhAHP *on salt tolerance of *D. hansenii*, the silencing and overexpression transformants were examined for their ability to grow on YM11 medium containing 2.5 M and 3.5 M NaCl, respectively. As demonstrated by real-time PCR, the RNAi transformant of *D. hansenii *exhibited reduced expression of *DhAHP *transcript in the presence of 2.5 M NaCl, relative to its wild type strain (Fig. 6A). Without any salt, both wild type strain and RNAi transformant showed a normal growth trend over 60 h (Fig. 6B). However, growth of the RNAi transformant was severely inhibited by 2.5 M NaCl.

**Figure 7:**
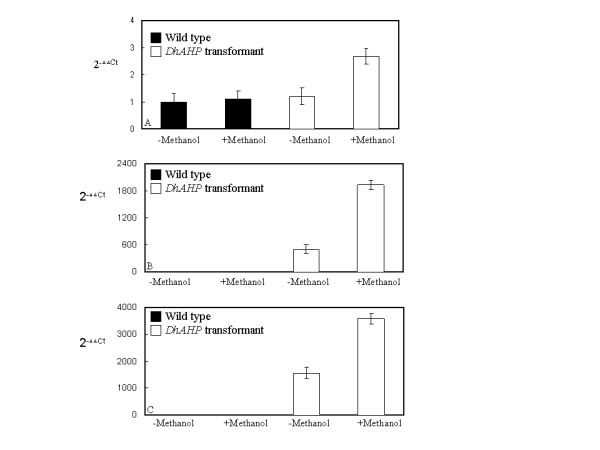
**Relative levels of *DhAHP *transcript of three yeasts and their *DhAHP *overexpression transformants**. Cells of *D. hansenii *(**A**), *S. cerevisiae *(**B**) and *P. methanolica *(**C**) were grown in media containing 3.5, 2.0 and 2.5 M NaCl, respectively, in the presence or absence of methanol for 72 min, and their *DhAHP *transcripts determined by real-time RT-PCR. For each species, the level for the wild type strain grown in media without methanol was taken as 1. Since the wild type strains of *S.c*. and *P.m *do not contain *DhAHP *their *DhAHP *transcript levels were low while their overexpression transformants showed high levels of expression relatively. Data presented were means +/- S.D. from 3–4 replicates of measurement.

**Figure 8:**
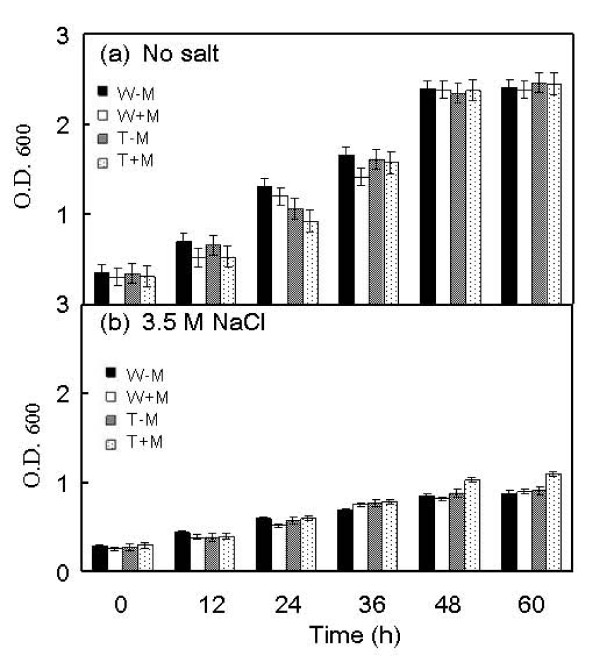
**Growth of *D. hansenii *and its *DhAHP *overexpression transformant as affected by salt**. Cells were cultured in YM11 media with or without 3.5 M NaCl and in the presence or absence of methanol for 5 days. W-M: wild type strain, without methanol, W+M: wild type strain, with 0.5% methanol, T-M: transformant, without methanol, T+M: transformant with 0.5% methanol. Data presented were means +/- S.D. from 3–4 replicates of measurement.

The overexpression transformant of *D. hansenii *had much higher *AHP *expression levels than its wild type counterpart when grown under 3.5 M NaCl and in the presence of the inducer methanol (Fig. 7A). Without any salt the overexpression trasnsformant showed a comparable growth to that of the wild type strain with or without the presence of methanol in the culture media (Fig. 8). Growth of both the wild type strain and the overexpression transformant was inhibited by 3.5 M NaCl (Fig. 8B).  However, only the overexpression transformant showed enhanced growth in the presence of the inducer methanol. Thus, overexpression and suppression of *DhAHP *reduce the salt tolerance of *D. hansenii*, respectively. The small enhancements in growth in the overexpression transformant under high salt, as compared to the wild type strain, is expected as expression of endogenous *DhAHP *can be largely induced by salt in this halophilic organism (Fig. 5).

### Overexpression of *DhAHP *in *S. cerevisiae *and *P. methanolica*

**Figure 9:**
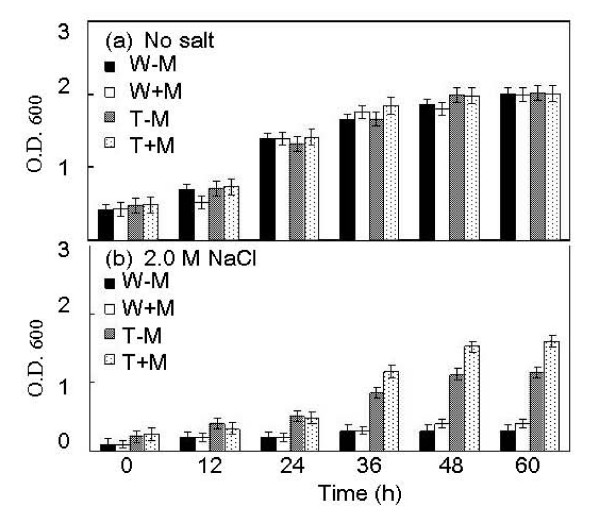
**Growth of *S. cerevisiae *and its *DhAHP *overexpression transformant as affected by salt**. Cells were cultured on YPD media with or without 2.0 M NaCl and in the presence or absence of methanol for 5 days. W-M: wild type strain, without methanol, W+M: wild type strain, with 0.5% methanol; T-M: transformant, without methanol; T+M: transformant with 0.5% methanol. Data presented were means +/- S.D. from 3–4 replicates of measurement.

**Figure 10:**
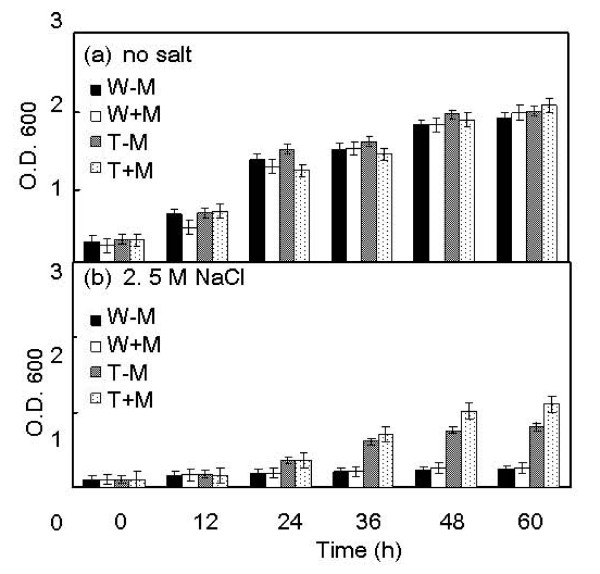
**Growth of *P. methanolica *and its *DhAHP *overexpression transformant as affected by salt**. Cells were cultured in YPAD media with or without 2.5 M NaCl and in the presence or absence of methanol for 5 days. W-M: wild type strain, without methanol, W+M: wild type strain, with 0.5% methanol; T-M: transformant, without methanol; T+M: transformant with 0.5% methanol. Data presented were means +/- S.D. from 3–4 replicates of measurement.

The function of *DhAHP *was further tested by overexpression of the gene in the two salt-sensitive yeasts *S. cerevisiae *and *P. methanolica*. As expected, the levels of *DhAHP *transcript in the wild type strains of the two species were very low even under high salt conditions, but its expression levels in the overexpression transformants increased drastically, especially in the presence of the inducer methanol (Figs. 7B, 7C). The salt tolerance of the overexpression transformants of the two yeasts was evaluated by culture in YPD medium containing 2.0 M NaCl for *S. cerevisiae *(Fig. 9b) and in YPAD medium containing 2.5 M NaCl for *P. methanolica*, relative to those of their wild type counterparts (Fig. 10b). For both species, the wild type strain and the overexpression transformant showed a normal and comparable growth over time when they were grown in media without containing salt in the presence or absence of methanol (Figs. 9a, Fig. 10a). In contrast, growth of the wild type strains of these salt-sensitive species was largely inhibited by high salt (Figs. 9b, Fig. 10b). However, only the overexpression transformants were able to maintain substantial growth under high salt, especially in the presence of methanol. The degrees of enhancement in salt tolerance by overexpression of *DhAHP *were more significant in *S. cerevisiae *and in *P. methanolica *(Figs. 9b, 10b) than in *D. hansenii *(Fig. 8b). The results indicate that overexpression of *DhAHP *confers enhanced salt tolerance to both salt sensitive *S. cerevisiae *and *P. methanolica*, allowing them to be able to grow at higher salt levels than they can normally tolerate.

### Intracellular ROS

**Figure 11:**
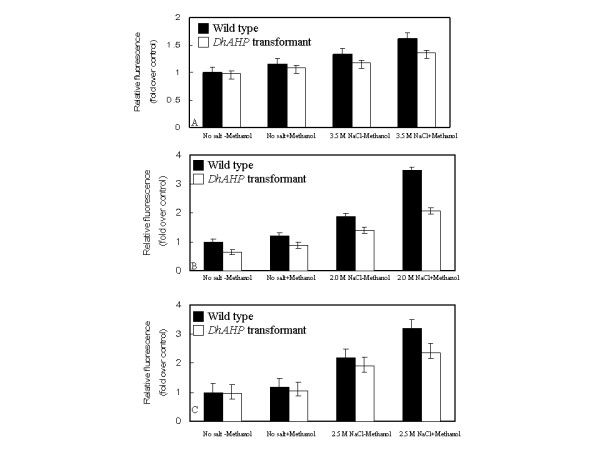
**Cellular ROS levels of three yeasts and their *DhAHP *overexpression transformants as affected by salt**. Cells of *D. hansenii *(**A**), *S. cerevisiae *(**B**) and *P. methonolica *(**C**) were grown in liquid media with or without salt and in the presence or absence of 0.5% methanol for 5 h. ROS levels, as measured by fluorescence signal, were presented as relative values. Data presented were means +/- S.D. from 3–4 replicates of measurement.

To see if the enhanced salt tolerance by overexpression of *DhAHP *in the three yeast species was due to reduced oxidative stress, the cellular ROS level was determined after the cells were grown under high NaCl conditions (3.5 M for *D. hansenii*, 2.0 M for *S. cerevisiae *and 2.5 M for *P. methanolica*) for 5 h. As shown in Fig.11A–C, NaCl induced accumulation of ROS in the wild type strains of the three yeast species, and the addition of methanol further increased its accumulation. It is also noticeable that the increases in ROS accumulation under high salt were much greater in *S. cerevisiae *and *P. methanolica *than in *D. hansenii*. The *DhAHP *overexpression transformants of the three species also exhibited a similar trend towards salt and methanol treatments but the amounts of ROS accumulated were considerably lower than those of their wild type counterparts. The reduction in ROS accumulation was more significant upon methanol induction, especially in the overexpression transformants of *S. cerevisiae *and *P. methanolica*. These results, correlated well with the data on levels of *DhAHP *expression (Fig. 7A–C) and on growth (Figs. 8, 9, 10), indicate that expression of *DhAHP *in these yeasts can lead to enhanced salt tolerance by reducing the level of accumulated ROS via *Dh*Ahp. Thus, the higher sensitivities of *S. cerevisiae *and *P. methanolica *to salt, relative to *D. hansenii*, may be associated with their inability to scavenge ROS.

## Discussion

Organisms are constantly exposed to various stresses, which cause considerable reduction in growth. In adaptation, organisms respond to stress through a number of physiological and developmental changes. Thus, expression of many genes is altered in such responses. Identification of the particular gene or genes responsible for the specific adaption to such stimuli is a major challenge in modern biology; it requires methods which rapidly and efficiently compare the transcripts expressed in the organism subject to stress. An equalizing cDNA subtraction hybridization method provides the technical basis for such a comparison. It has been demonstrated successfully to clone a number of differentially expressed genes [27]. Isolation of differentially expressed genes in the extremely halophilic yeast *D. hansenii *would serve as an initial step towards understanding its tolerance mechanisms against salinity.

### Salt-induced genes in *D. hansenii*

As discussed in the Background section, a number of salt-related genes have been identified in the extremely halophilic yeast *D. hansenii*. As expected, most of the salt-upregulated genes identified so far are involved in osmoregulation or transport of ions. By using forward subtractive hybridization, we have identified, cloned and sequenced *DhAHP*, a new salt induced gene, from *D. hansenii *by applying salt stress. Further characterization of the functional role of the gene will aid to our understanding of the underlying halotolerance mechanisms in this halophilic yeast.

### Characterization of salt-induced *DhAHP *and its protein

High salinity, which is caused typically by NaCl, results in ion toxicity and hyperosmotic stress leading to numerous secondary pathological effects including generation of ROS [28] and programmed cell death. It's not surprising that one of the major upregulated genes under salinity stress, *DhAHP*, is orthologous to the alkyl hydroperoxide reductase of the peroxiredoxin family. Ahp is a member of the peroxiredoxin family of enzymes, which possess activity against H_2_O_2_, organic peroxides, and peroxynitrite [18]. *DhAHP *has not been previously described for its role in salt tolerance in *D. hansenii*. Comparison of protein sequences showed that *Dh*Ahp shares a high similarity to Ahp11 of the yeast *C. albicans*. Multiple sequence alignment analysis of Ahps showed the protein from *D. hansenii *has a high similarity to that of *C. albicans *(Fig. 3A) and phylogenetic analysis revealed that Ahp of *D. hansenii *is more closely related to the yeast than to the plant or mammalian peroxiredoxins (Fig. 3B). Thus, *Dh*Ahp belongs to the alkyl hydroperoxide reductase of the peroxiredoxin family. Previously, Kurtzman and Robnett [29] have suggested that *D. hansenii *is phylogenetically related to *C. albicans *based on the fact that they are both ascomycetous yeasts. The high similarity between the Ahps from both species further supports this notion. In addition, both organisms use an alternative genetic yeast code in which the CUG codon may be used as a serine codon [30]. Taken together, these results suggest that *Dh*Ahp and *C. albicans *Ahp11 have common ancestry, but show divergent evolution.

The closest structural homolog to *DhAHP *is the PrxD (Type Ii) of *Populus tremula *(PDB:1TP9A) (data not shown), which contains two cysteine residues. Though poplar Prx contains two conserved cysteine residues, it is assumed to function as a 1-Cys Prx because site-directed mutagenesis has demonstrated that only the catalytic cysteine of the poplar Prx is essential for hydroperoxide reduction [31]. Previously, the type II TPx from *S. cerevisiae *was reported to contain three Cys residues at positions 31, 62 and 120, and its disulfide linkage is between 62 and 120 and Cys-31 has no effect on TPx activity [32]. Though structural and sequence analyses of the deduced protein indicate that *Dh*Ahp contains 2 Cys residues at positions 24 and 54, the multiple sequence alignment of Ahps identifies the conserved Cys-54 as the peroxidative cysteine (Fig. 3). The role of Cys-24 in *D. hansenii *Ahp remains to be explored in the future. Therefore, *Dh*Ahp is clearly a member of the disulfide oxidoreductases and can be considered a 1-Cys Prx.

### Regulation of expression of *DhAHP*

Alkyl hydroperoxide reductases have been identified previously as oxidative stress proteins in *Salmonella typhimurium *[33] and *Bacillus subtilis *[23] and their expression is known to be upregulated by oxidative factors. However, the finding of an extensive accumulation of Ahp in the halophilic yeast *D. hansenii *by salt is reported for the first time in this study. Consistently, overexpression of *D. hansenii *Ahp in *D. hansenii *(Fig. 7) and in the two salt-sensitive yeasts *S. cerevisiae *and *P. methanolica *(Fig. 8 and 9) further increases their tolerance to salt. On the contrary, suppression of its expression in *D. hansenii *resulted in a lower tolerance to salinity (Fig. 6). Clearly, the results suggest that *DhAHP *is induced by salt and its expression confers the high salt tolerance in *D. hansenii*. A previous study also revealed that the expression of a homolog to the *Escherichia coli *Ahp is induced by osmotic shock in *Staphylococcus aureus *[34]. Similarly, the expression of Ahp in *Shewanella putrefaciens *is accompanied by accumulation of the corresponding transcript under NaCl stress [35] and the activity of the alkyl hydroperoxidase enzyme is dependent on salt concentration [36]. Collectively, the results from these studies indicate that expression of Ahps in general is upregulated not only by oxidative factors but also by other stresses, such as drought and salinity.

Hydrogen peroxide level is known to increase within the cell in response to various stress factors and act as an intracellular messenger for induction of genes related to defense against oxidative environments [37]. Treatment of cells with hydrogen peroxide mimics stress and induces defense signaling by activating mitogen-activated protein kinase and stimulates cell growth [38]. The ROS levels of *D. hansenii*, *S. cerevisiae *and *P. methanolica *also increase in response to salt and methanol treatments, and the degrees of increase are more pronounced in the two salt-sensitive yeast species than the halophilic *D. hansenii *(Fig. 11). Furthermore, the *DhAHP *overexpression transformants of these species have reduced amounts of ROS accumulated than their wild type strains, indicating the protective role of Ahp. These results are in agreement with the earlier observations that *Ahp *genes play an important role in peroxide resistance in *Bacillus subtilis *[23], *Clostridium pasteurianum *[24], *Burkholderia cenocepacia *[25], *Shewanella putrefaciens *[35] and *Porphyromonas gingivalis *[39] under various stress conditions (e.g. hydrogen peroxide, high/low temperature and high/low pH). Therefore, the induced expression and accumulation of *Dh*Ahp in saline environments to detoxify ROS is a very important survival mechanism for this halophilic organism.

## Conclusion

In summary, the *Ahp *gene isolated from the extremely halophilic yeast *D. hansenii *under salt stress in this study is a new gene relative to its salt tolerance mechanism. It is rapidly induced and accumulates to large quantities in *D. hansenii *to reduce accumulation of ROS. Molecular characterization shows that *Dh*Ahp, a cytosolic protein, belongs to the alkyl hydroperoxide reductase of the 1-Cys type peroxiredoxin family. The *Dh*Ahp and *C. albicans *Ahp11 have a common ancestry but show divergent evolution. Silencing of its expression by RNA interference resulted in decreased tolerance to salt stress. On the other hand, overexpression of the *DhAHP *in *D. hansenii *and the two salt-sensitive yeasts *S. cerevisiae *and *P. methanolica *conferred enhanced tolerance to salt with reduced accumulation of ROS. Clearly, the multiple activities (peroxidase, chaperone, redox signaling) possessed by Ahps are essential for its central role in protecting the cellular metabolism of yeast against ROS built-up under stress conditions. Compared with the two salt-sensitive yeasts, the extreme halotolerance exhibited by *D. hansenii *may be due to its ability to scavenge ROS by Ahp. Thus, the results of this study contribute to our understanding of the underlying mechanisms by which the extremely halophilic yeast *D. hansenii *adapts to high salt. Manipulation of antioxidant enzymes in industrial microorganisms, as demonstrated in *S. cerevisiae *and *P. methanolica *in this study, or crops may bring about enhanced growth and production of useful products under adverse culture conditions. Overexpressing enzymes involved in redox reaction in crops, such as superoxide dismutase [40] and glutathione peroxidase [41] has resulted in enhanced tolerance to salt and other stress.

## Methods

### Yeast strains and growth conditions

The yeast strains used in this work included *D. hansenii *strain BCRC No. 21947, isolated from Hsilo County, Taiwan, *S. cerevisiae *Neo Type strain Y1 BCRC No. 21447 from brewer's top yeast, obtained from FIRDI (Food Industry Research and Development Institute, Hsin-chu City, Taiwan), and *P. methanolica *strain PMAD11 genotype *ade2-11*, obtained from Invitrogen, U.S.A. *D. hansenii *was cultured at 24°C in YM medium (0.3% yeast extract, 0.3% malt extract, 0.5% peptone, 1% dextrose) while *S. cerevisiae *and *P. methanolica *were cultured at 28°C in YPD medium (1% yeast extract, 2% peptone, 2% dextrose) and YPAD medium (1% yeast extract, 2% peptone, 2% dextrose, 0.01% adenine), respectively.

### RNA extraction and poly(A^+^) RNA purification

Total RNA was extracted with a modified hot phenol protocol [42]. Poly (A^+^) RNA was isolated from total RNA using Mag-Net mRNA Isolation Kit according to the manufacturer's instruction (Amresco, Inc. USA). Concentration of RNA was determined using a NanoDrop spectrophotometer (NanoDrop, Wilmington, USA). RNA quality was verified by electrophoresis on 1.5% formaldehyde agarose gel and stained with ethidium bromide.

### Subtractive hybridization and construction of subtracted cDNA library

Subtractive hybridization was performed using PCR-select cDNA Subtraction Kit (Clontech, Palo Alto, CA, U.S.A.). For screening of differentially upregulated genes, cDNA synthesized from the 2.5 M NaCl treated yeast cells for 24 min was used as the tester while that from non-treated cells served as the driver. The PCR products of forward subtraction were subcloned into the pGEMR-T Easy Vector (Promega, USA). Competent cells of *E. coli *(XL-Blue) was transfected with the plasmids and grown on LB-agar medium containing 5-bromo-4-chloro-3-indolyl-b-d-galactoside (X-gal) (Sigma, U.S.A.), isopropyl β-D-1-thiogalactopyranoside (IPTG) (Sigma, U.S.A.) and ampicillin. Individual white colonies with insert DNA were randomly picked for further analysis.

### Sequencing and sequence analysis

White clones from the forward subtractive hybridization libraries were sequenced with the universal T7 or SP6 sequencing primers using an automatic DNA sequencer (3100 Genetic Analyzer, ABI, U.S.A). All inserted sequences were queried for similarity through the NCBI database using BLASTX sequence comparison software http://www.ncbi.nlm.nih.gov/BLAST.

### Quantification of *DhAHP *by quantitative real-time PCR (Q-RT-PCR)

Total RNA isolated from yeast cells treated with NaCl for various time intervals was first treated with DNase I (Promega, U.S.A.) to remove DNA contamination before cDNA synthesis [43]. cDNA was synthesized using High Capacity cDNA Reverse Transcription Kit (P/N 4368814, ABI, U.S.A.) for RT-PCR according to the manufacturer's instruction. The sequence forward and reverse primers for Q-RT-PCR were designed using the primer Express^R ^Software provided by Applied Biosystems. A set of *D. hansenii *18S ribosomal RNA primers was designed for use as an endogenous control.

18S forward: G'-CGTCCCTGCCCTTTGTACAC-3'

18S reverse: G5'-GCCTCACTAAGCCATTCAATCG-3'

*DhAHP *target forward: G5'-GGAGCCCCAGGAGCATTTA-3'

*DhAHP *target reverse: G5'-TGGGCCAAATAATCGGGAAT-3'

Real-time PCR assay was carried out in an ABI PRISM 7500 Sequence Detection System (ABI, U.S.A.). The amplification of the target genes was monitored every cycle by SYBR-Green fluorescence.

### Rapid amplification of cDNA ends (RACE)

The full-lengthed cDNA clone of *DhAHP *was obtained by rapid amplification of the cDNA ends using the GeneRacerTM Kit (Invitrogen, U.S.A.), as described in the manual provided by the manufacturer. The forward and reverse gene specific primers (GSPs) used for RACE were designed based on the *DhAHP *cDNA sequence. The universal primers for 5' and 3' Race were GeneRace 5' and GeneRace 3', respectively, provided in the kit. After PCR the DNA fragments were cloned into pGEMR-T Easy vector (Promega, U.S.A.) for sequencing.

Forward (GSP): 5'- GTCAATGCTGCTTGGGGTAAAGCTTTA-3'

Reverse (GSP):5'- GGTCTCAGCACTGGAAATTTCAGTG-3'

GeneRace 5':5'- CGACTGGAGCACGAGGACACTGA-3'

GeneRace 3':5'- GCTGTCAACGATACGCTACGTAACG-3'

### Bioinformatics analysis

The deduced amino acid sequence of *DhAHP *was analyzed with the Expert Protein Analysis System http://www.expasy.org/. Multiple sequence alignment was performed for sequence comparison and alignment of *D. hansenii *Ahp and two other reported AHPs (Swiss-Prot: P38013 and Q5AF44) from *S. cerevisiae *and *C. albicans *and peroxisomal membrane protein (Swiss-Prot: O14313) from *S. pombe *and three other structural homolog proteins (Swiss-Prot:Q8S3L0, B3GV28 and P30044) from *P. tremula*, *P. sativum *and *H. sapiens*. The alignment and phylogenetic analysis were carried out by the protein sequence alignment program CLUSTAL W.

### Southern and northern hybridization analysis

Genomic DNA was isolated from yeast cells by the method of Hoffman and Winston [44]. Southern and northern hybridization analyses were performed using the DIG High Prime DNA Labeling and Detection Starter Kit (Roche Diagnostics, Switzerland). For Southern hybridization, 20 μg genomic DNA was digested with *Eco*RI and *Bam*HI and electrophoretically separated on 0.7% (w/v) agarose gels in TBE buffer and DNA fragments blotted onto nylon membrane (Amersham Pharmacia Biotech, U.K.) by 20×SSC. The full-lengthed *DhAHP *DNA was labeled and used as a hybridization probe. For nothern hybridization analysis, RNA was extracted from *D. hansenii *that was not treated or treated with 2.5 M NaCl for 16 min, separated by electrophoresis in formaldehyde gels, blotted onto nylon membrane and hybridized with labeled *DhAHP *DNA, as described above for Southern analysis.

### Silencing of *DhAHP *expression in *D. hansenii *by RNA interference

To test the function of *DhAHP*, RNA interference was employed to suppress its expression in *D. hansenii *using the Knockout RNAi System Kit (Clontech, U.S.A.), as described in the manual by the manufacturer. The oligonucleotide sequences including *Bam*HI and *Eco*RI sites, target sense sequence, hairpin loop, target antisense sequence and terminator were shown as follow.

BamHI Target sense sequence Hairpin loop Target antisense sequence Terminator

**5'**-GATTCGACATATTMLCGATTATTTGTTCAAGAGACAAATAATCGGGAATATGTTTTTTTG-3'

**3'**-GCTGTATAAGGGCTAATAAACAAGTTCTCTGTTTATTAGCCCTTATACAAAAAAACTTAA-3' *EcoR*I

A chemical method based on LiCl, as described by Tarutina and Tolstorukov [45], was used to transfect *D. hansenii *and the RNAi transformant was screened by its poor ability to grow on YM11 solid media containing 2.5 M NaCl. The transformant was confirmed by sequencing the introduced DNA fragment in the genome with specific primers and by Q-RT-PCR.

### Overexpression of *DhAHP *in *D. hansenii*, *S. cerevisiae *and *P. methanolica*

To further test its functional role in relation to salt tolerance, *DhAHP *was overexpressed in three yeast species with contrasting degrees of salt tolerance. The entire ORF of *DhAHP *was first amplified by PCR utilizing the overexpression 5' primer, which introduced an *Eco*RI site in front of the starting ATG codon, and the overepression 3' primer, which introduced a *Bam*HI site before the stop codon. This DNA fragment was inserted into the expression vector of pMETB (Invitrogen, U.S.A.). The plasmid DNA of the *DhAHP/*pMETB veector was digested with *Pst *I to release the *P*_*AUG1*_/*DhAHP *expression cassette, which was then introduced into *D. hansenii*, *S. cerevisiae *and *P. methanolica *by a chemical method based on LiCl, as described by Tarutina and Tolstorukov [45]. The AUG1 sequence is a methanol inducible promoter to drive the expression of introduced *DhAHP*. Functional complementation was used to screen transformants from the three species by culture on solid media containing 0.5% methanol and higher NaCl concentrations than they can normally tolerate. For isolation of *D. hansenii *overexpression transformants YM medium containing 3.5 M NaCl was used, for *S. cerevisiae *transformants YPD medium containing 1.5 M NaCl was used and for *P. methanolica *transformants YPAD medium containing 2.0 M NaCl was adopted. The transformants were confirmed by sequencing the ***P***_*AUG1 *_DNA fragment in the genome with specific primers and by Q-RT-PCR with cells grown under high salt in the presence or absence of methanol. The ability of the selected transformants to tolerate salt was further assessed by growing in liquid media containing high NaCl concentrations.

### Measurement of intracellular ROS

For measurement of cellular ROS, the redox-sensitive fluorescent probe 2', 7'-dihydrodichlorofluorescein diacetate (DCFA-DA) (Sigma, U.S.A.) was used according to Chattopadhyay *et al*. [46]. The cells of wild type strains and *DhAHP *overexpression transformants were grown in appropriate liquid media without any salt for approximately 36 h (1 O.D. at 600 nm) and switched to fresh media containing high NaCl (3.5 M for *D. hansenii*, 2.0 M for *S. cerevisiae *and 2.5 M for *P. methanolica*) with or without methanol for 5 h. To determine ROS, cells were harvested by centrifugation and treated with 10 μM DCFA for 30 min at 30°C. The cells were re-suspended and washed in water and extracted by vortexing with glass beads. Extracts were centrifuged and fluorescence in the supernatant was measured with *λ*EX = 485 nm and *λ*EM = 524 nm in a fluorescence spectrophotometer (Infinite F200). Fluorescence signals were expressed relative to that of the wild type strain before any stress treatments (fold over control).

## Authors' contributions

HFC planned and designed the study, performed the experiments and analyzed the results and drafted the manuscript. YFY contributed equally. MSBK initiated and supervised the study, assisted in data analysis and revised the manuscript. All authors read and approved the final manuscript.
